# A high content clonogenic survival drug screening identifies maytansine as a potent radiosensitizer for meningiomas

**DOI:** 10.3389/fimmu.2025.1557165

**Published:** 2025-03-18

**Authors:** Jinxiu Yu, Jiaojiao Deng, Leihao Ren, Lingyang Hua, Tianqi Wu, Yi Hui, Chunlin Shao, Ye Gong

**Affiliations:** ^1^ Department of Neurosurgery, Huashan Hospital, Shanghai Medical College, Fudan University, Shanghai, China; ^2^ Institute of Neurosurgery, Fudan University, Shanghai, China; ^3^ Shanghai Key Laboratory of Brain Function Restoration and Neural Regeneration, Fudan University, Shanghai, China; ^4^ Department of Radiotherapy, Huashan Hospital, Shanghai Medical College, Fudan University, Shanghai, China; ^5^ Translational Medical Center for Stem Cell Therapy, Shanghai East Hospital, School of Medicine, Tongji University, Shanghai, China; ^6^ Institute of Radiation Medicine, Shanghai Medical College, Fudan University, Shanghai, China; ^7^ Department of Critical Care Medicine, Huashan Hospital, Shanghai Medical College, Fudan University, Shanghai, China

**Keywords:** meningioma, radiosensitivity, radiotherapy, high content clonogenic survival drug screening, maytansin

## Abstract

**Purpose:**

Radiation resistance significantly hinders the efficacy of radiotherapy for meningiomas, posing a primary obstacle. The clinical inadequacy of therapeutic drugs and radiosensitizers for treating meningiomas further exacerbates the challenge. Therefore, the aim of this study was to identify potential radiosensitizers for treating meningiomas.

**Methods:**

A high content clonogenic survival drug screening was employed to evaluate 166 FDA-approved compounds across varied concentration ranges. Cell viability, apoptosis, and radiosensitization were assessed using CCK-8 assays, Annexin V-FITC/PI assays and standard colony formation assays. Transcriptome sequencing, immunofluorescence and cell cycle experiments were conducted to assess transcriptional profile, DNA double-strand break damage and cell cycle distribution. Finally, the radiosensitizing effect of Maytansine was assessed *in vivo* through subcutaneous tumor implantation in nude mice.

**Results:**

The proportion of maytansine exhibiting SRF≥1.5 within the detectable concentration range was 100%. CCK-8 assay indicated the IC50 values of maytansine for IOMM-Lee and CH157 were 0.26 ± 0.06 nM and 0.31 ± 0.01 nM, respectively. Standard clonogenic survival assays and Annexin V-FITC/PI assays revealed maytansine had a notable radiosensitizing effect on meningioma cells. Transcriptome sequencing analysis demonstrated that maytansine can modulate cell cycle and DNA damage repair. Immunofluorescence analysis of γ-H2AX and cell cycle experiments demonstrated that Maytansine enhances DNA double-strand breaks and induces G2/M phase arrest. Moreover, *in vivo* studies had indicated that Maytansine augments the therapeutic efficacy of radiotherapy.

**Conclusion:**

This study highlighted the potential of maytansine as a potent inhibitor and radiosensitizer for meningiomas by inducing G2/M phase cell cycle arrest and enhancing DNA double-strand break damage. These findings opened up a promising path in the development of radiosensitizers aimed at treating this condition.

## Introduction

Meningiomas are the most common intracranial tumors, accounting for approximately 39.7% of all intracranial tumors and about 55.4% of non-malignant intracranial tumors (1). In the United States (US), the incidence rates for meningiomas were 9.51 per 100,000 population among CBTRUS specific histopathology groupings (1). Meningiomas are more common in people over 65 years old and less common in those under 14 years old, with the incidence increasing with age. According to the WHO classification, meningiomas are categorized into WHO grades 1, 2, and 3. Of meningiomas with documented WHO grade, 80.1% of meningiomas are grade 1, about 18.3% are grade 2, and around 1.5% are grade 3 (1).

The treatment for intracranial meningiomas mainly consists of surgical resection and radiotherapy. When treatment is required for meningiomas, surgery is typically recommended as the first choice (2). The surgical principle for meningiomas remains to achieve maximal safe resection while preserving the integrity of adjacent normal brain structures as much as possible (2). However, Grade 2 and 3 meningiomas exhibit a tendency to recur, even in cases of aggressive treatment. Compared to benign meningiomas, a significant portion of high grade meningiomas exhibit more aggressive behavior, with a recurrence risk increased by approximately 6 to 8 times, and the risk of dying from tumor progression is significantly higher (3, 4). Previous research reports had indicated that for grade 2 meningiomas, the 5-year progression-free survival rates (PFS) after gross total resection (GTR) and subtotal resection (STR) were 70% and 60% respectively (5, 6). For grade 3 meningiomas, the 5-year PFS was between 12% to 57%, even after undergoing surgical resection and radiotherapy (7, 8).

In addition to surgery, it is generally recommended to administer external beam radiotherapy after resection of grade 2 and grade 3 meningiomas to increase local control (2). For grade 2 meningiomas, the 5-year PFS after radiotherapy ranged from 47.4% to 82.6% in most studies (6, 9–14). For grade 3 meningiomas, the 5-year overall survival (OS) ranged from 19% to 61% (8, 15–17). Due to the inherent radio-resistance of meningiomas, the effectiveness of radiotherapy is limited. Additionally, patients who have previously undergone radiotherapy tend to have poorer outcomes (18). Furthermore, the number of recurrences is also an adverse prognostic factor that affects treatment outcomes. With each successive recurrence, the likelihood of achieving durable local control of atypical meningiomas through further salvage treatments decreases (18). For patients who are not appropriate candidates for further surgical or radiotherapy, there is currently no effective treatment option. Thus, there is an urgent imperative to advance novel therapies for radioresistant tumors. Since radiation therapy serves as a critical adjunctive treatment alongside surgery, incorporating radiosensitizers to enhance its effectiveness emerges as a compelling strategy.

In this study, we adopted high-content clonogenic survival screen to identify potential radiosensitizers for meningiomas. A library of FDA approved compounds was tested against meningioma cell followed by high-content imaging to assess the effect of radiotherapy sensitization. The screen successfully identified a potent radiosensitizer for meningioma.

## Materials and methods

### Cell culture

The IOMM-Lee and CH157-MN meningioma cell lines were generously provided by Prof. Wan’s lab at the H. Lee Moffitt Cancer Center in Tampa, FL. The meningioma cells were maintained in recommended DMEM/F12 medium (Gibco, 11320033) supplemented with 10% fetal bovine serum (FBS, Sigma, F8192) and 1% penicillin/streptomycin (Invitrogen) at 37°C in a 5% carbon dioxide cell culture incubator. Before use in experiments, all cell lines were tested and confirmed negative for mycoplasma contamination.

### Reagents and irradiator

A library of FDA-approved compounds, including maytansine, was purchased from MedChemExpress. Meningioma cells were irradiated at a dose rate of 0.883 Gy/min using an X-ray irradiator (XRAD320, PXI Inc., North Branford, CT, USA) operating at 320 kV and 12.5 mA. The irradiator undergoes annual calibration to ensure accuracy.

### High-content clonogenic survival assay

The IOMM-Lee cell line was utilized for a high-content clonogenic survival assay. A single-cell suspension, containing 50 cells per well in triplicate and with a total volume of 90 microliters per well, was seeded into standard 96-well tissue culture plates (Thermo Scientific Nunc). Subsequently, the plates were placed in a humidified cell culture incubator at 37°C and 5% CO_2_ overnight after seeding. Seven concentration gradients were established, and a 10-fold final concentration of the drug, at 10 µl per well, was added to the plates, resulting in final concentrations of (10 µM, 1 µM, 100 nM, 10 nM, 1 nM, 100 pM, and 10 pM). After a 4-hour incubation with the drugs, the cells were exposed to 2 Gy radiation using the XRAD320 irradiator. Upon completion of irradiation, the plates were returned to the incubator at 37°C and 5% CO_2_ for an additional 7-10 days. After fixation and staining with crystal violet, colonies were automatically counted using ImageXpress Micro 4 (Molecular Devices).

### Flow cytometry cell cycle analysis

A 6-well plate was seeded with 100,000 cells per well and incubated overnight. The cells were then treated with drugs or 0.1% DMSO, followed by ionizing radiation (IR) or sham irradiation. After 24 hours, the cells were detached using trypsin, resuspended in 1 mL of culture medium, and centrifuged at 300 × g and 4°C for 5 minutes followed by the addition of 1 mL of pre-chilled phosphate-buffered saline (PBS) and another round of centrifugation. This was succeeded by the addition of 1 mL of pre-chilled 70% ethanol, fixing the cells at 4°C for 24 hours. The cells were then centrifuged again at approximately 1000 × g for 5 minutes, then washed with 1 mL of pre-chilled PBS and centrifuged once more. Finally, 0.5 mL of staining mixture (composed of 0.5 μL staining buffer solution, 10 μL RNase A (50X), and 25 μL propidium iodide (PI) (20X)) was added to each sample, followed by incubation in the dark at 37°C for 30 minutes and subsequent analysis using flow cytometry.

### Quantification of apoptosis via annexin V-FITC/PI staining

A 6-well plate was seeded with 100000 cells per well and incubated overnight. The cells were treated with drugs or 0.1% DMSO, followed by IR or sham irradiation. After 48 hours, the cells were harvested, washed with pre-chilled PBS, and resuspended. After centrifugation to remove the supernatant, Annexin V-FITC/PI was added and incubated in the dark for 5 minutes, and subsequently analyzed using Flow Cytometry.

### CCK8 cell viability assay

The cells were seeded at a concentration of 2 × 10^3 cells/mL on a 96-well plate and treated with varying doses of maytansine for 72 hours. Subsequently, incubate the cells with 10 µl of CCK8 for 2 hours, followed by the measurement of the absorbance of the samples at 450 nm using a microplate reader. The IC50 values were determined using the Logit method.

### Clonogenic assay

To prepare a single-cell suspension, cells in the logarithmic growth phase were harvested using 0.1% trypsin/EDTA. A suitable number of cells were seeded in triplicates in a 6-well plate according to different irradiation doses, with 3 mL of culture medium added to each well for cultivation. After seeding the cells, they were incubated overnight in an incubator. The cells were treated with a drug at an IC30 final concentration, and irradiation was performed 4 hours after drug treatment. After 72 hours, the drug-containing medium was replaced with complete medium without drugs, and incubation continued for another 10-14 days. The colonies (with more than 50 cells) were fixed with 4% paraformaldehyde, stained with 0.5% crystal violet for 20 minutes, and then washed, dried, and the colonies were counted.

### Immunofluorescent γH2AX DNA damage assay

Immunofluorescence staining for γ-H2AX was conducted. Cells were seeded in 24-well plates at a density of 1×10^4^ cells per well and cultured with 1 milliliter of complete medium overnight in a cell culture incubator. Subsequently, the cells were treated with drugs or 0.1% DMSO for 4 hours, followed by exposure to IR or sham irradiation. The cells were fixed with 4% paraformaldehyde, washed thrice with PBS, permeabilized with 1% Triton X-100, then blocked with 1% bovine serum albumin (BSA). Rabbit primary antibodies against γ-H2AX (CST) were applied at a 1:250 dilution for staining and incubated at 4°C overnight. After 3 times PBS washes, Alexa Fluor 488-conjugated secondary antibodies (Absin) were added at a 1:1000 ratio for staining and incubated at room temperature for 1 hour. Following 3 times PBS washes, the cell nuclei were stained with DAPI (Beyotime). Images were captured using the ImageXpress Micro 4 (Molecular Devices), and quantitative analysis of γ-H2AX foci was performed in 50 cells.

### RNA-sequencing analysis

After 24 hours of drug treatment on the cells, total RNA was extracted and sequenced on Illumina HiSeq™ platform. The read data underwent quality check using FastQC (version 0.11.2) and were mapped to the human reference (GRCh38, gene annotation GENCODE version 30) using HISAT2 (version 2.1.0) with default settings. Gene expression was evaluated using StringTie and known gene models. Differential gene expression was determined using DESeq2. Functional enrichment analysis was performed using DAVID to predict the association of the altered genes with disease phenotypes.

### *In Vivo* studies

Six-week-old athymic nude mice were obtained from GemPharmatech Co. Ltd. IOMM-Lee cells (1×10^6) were subcutaneously injected into the right flank of the mice. Upon the tumor size reaching around 100 mm³, the mice were randomly divided into four groups (1): i.v. injected with PBS (2); i.v. injected with Maytansine 0.8mg/kg (3); IR 7.5 Gy (4); i.v. injected with Maytansine 0.8mg/kg + IR 7.5 Gy.

Only the tumor sites were exposed to the radiation, while the remainder of the mice’s bodies were shielded with lead plates. The tumor volume was calculated using the standard formula V = L*W^2/(π/6) (L=length, W=width). Measurements of tumor volume were taken every three days. When the tumor volume approached 1000 mm^3^, the mice were euthanized using cervical dislocation. The xenograft tumors were then excised, photographed, and divided into two halves: one half was fixed and embedded in paraffin for immunofluorescence staining, while the other half was rapidly frozen in liquid nitrogen for storage. All procedures involving nude mice were performed in accordance with the “Animal Research: Reporting of *In Vivo* Experiments” (ARRIVE) guidelines and the Guide for the Care and Use of Laboratory Animals published by the US National Institutes of Health (NIH publication No. 8023, revised in 1978).

### Data analysis

A colony was considered a colony only if it contains 50 or more cells. For each test condition, calculated the SRF_2Gy+Drug_ value according to the following formula (20):


$${\text{SRF}}_{2\text{Gy}+\text{Drug}}=\;\left({\text{NCN}}_{2Gy}{\text{x NCN}}_{\text{at concentration x}}\right)/{\text{NCN}}_{2Gy+Drug at concentration x}$$

NCN = normalized colony number relative to control;

$${\text{NCN}}_{2\text{Gy}}=\text{average colony number of }2\text{ Gy}/\text{average colony number of control}$$

$${\text{NCN}}_{\text{Drug at concentration x}}=\text{ average colony number of drug at concentration x}/\text{average colony number of control}$$

$${\text{NCN}}_{2Gy+Drug at concentration x}=\;average colony number of 2Gy+\text{Drug at concentration x}/\text{average colony number of control}$$

### Statistical analysis

All data were presented as mean ± SEM. One-way analysis of variance (ANOVA), two-way ANOVA, and t-tests were performed for statistical analysis using Graph Pad Prism 9 software. A p-value less than 0.05 was considered statistically significant and denoted by “*”.

## Results

### A high-content clonogenic survival drug screening identified maytansine as a potent radiosensitizer for meningiomas

**Figure 1:**
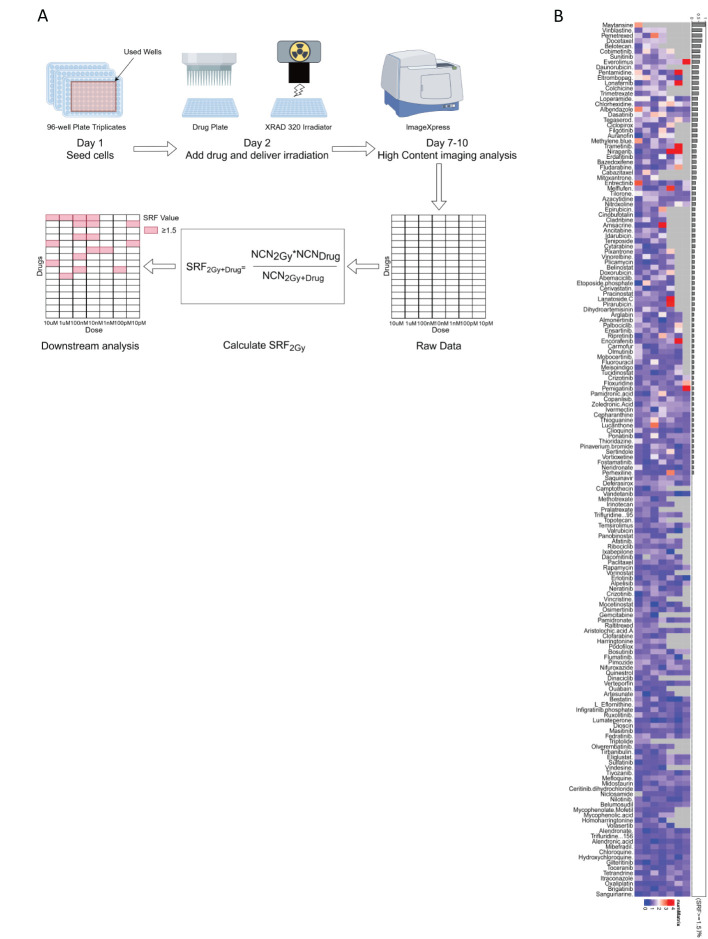
**(A)** The workflow for the high-content clonogenic survival drug screening process aimed to identify radiosensitizers for meningiomas. **(B)** The heatmaps depicted the SRF values for the IOMM-Lee cell line at different concentrations, organized based on (SRF ≥ 1.5)%.

In this study, 166 custom compounds were chosen for a high-content clonogenic survival drug screening. Due to the larger size of the colonies formed by CH157 cells, they were deemed unsuitable for the screening in a 96-well plate. As a result, IOMM-Lee cells were selected for the high-content clonogenic survival drug screening on a 96-well plate. The research workflow was illustrated in **Figure 1A** (1): IOMM-Lee cells were initially seeded at 50 cells per well on the first day (2); Following overnight incubation, the cells were exposed to various drug concentrations (10 µM, 1 µM, 100 nM, 10 nM, 1 nM, 100 pM, and 10 pM) and irradiated with 2 Gy four hours later; (3) Post-irradiation, the cells were incubated for 7-10 days, then fixed, stained with crystal violet, and colonies containing 50 cells or more were quantified using a high-content imaging system; (4) The data analysis included determining the SRF values for each drug at different concentrations, subsequent data visualization, and downstream analysis.

Certain drugs can inhibit IOMM-Lee cells, potentially causing cell death at high concentrations and making the SRF value unavailable. Among the 166 compounds studied, maytansine was found to be strongly cytotoxic to IOMM-Lee cells at concentrations ranging from 10 uM to 100 pM, preventing the determination of SRF values. The proportion of maytansine exhibiting SRF values of at least 1.5 within the detectable concentration range was 100% (**Figure 1B**). The significant toxicity of maytansine towards IOMM-Lee cells and its radiosensitizing properties led to its identification as a potent radiosensitizing drug for meningiomas in this study.

### Maytansine exhibited inhibitory effects on meningioma cells and enhanced sensitivity to IR *in vitro*

**Figure 2:**
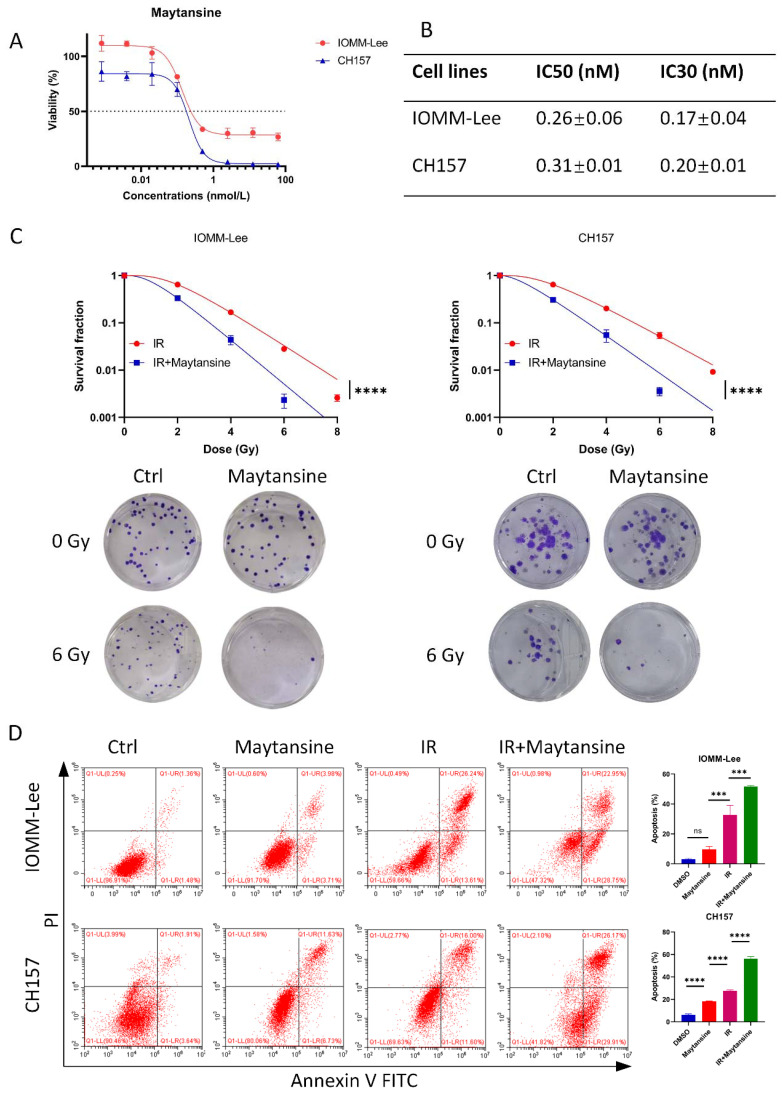
Maytansine inhibited meningioma cell viability and enhances radiotherapy sensitivity in vitro. A-B. Effect of maytansine on the viability of meningioma cells. Cells were challenged with increasing concentrations of maytansine for 72 h and cell viability was measured by CCK8. IC50 and IC30 values in IOMM-Lee and CH157 cell lines were shown. C. The effects of Maytansine on radiosensitivity of IOMM-Lee and CH157 were demonstrated by clonogenic assay. Maytansine at the IC30 concentration was added to each cell 4 hours prior to irradiation. Subsequently, after 10-14 days of seeding, clones consisting of 50 or more cells were counted. Survival curve was then constructed based on the Linear Quadratic Model. D. Cell apoptosis was evaluated in four groups: control, maytansine, IR, and Maytansine+IR. After a 48-hour treatment, Annexin V FITC/PI assay was utilized to measure apoptosis, demonstrating a higher rate in the combined therapy group than in monotherapy groups. The data represented the mean ± SD from three independent experiments. Statistical significance was assessed using a two-tailed Student’s t-test and two-way ANOVA. Significance levels were denoted as *** p< 0.001, and **** p< 0.0001.

To assess the efficacy of maytansine, we investigated its effects on the cell viability of t IOMM-Lee and CH157 meningioma cell lines, *in vitro*. Cell viability was assessed using CCK8 assays following 72 hours of maytansine treatment. The results demonstrated a notable, dose-dependent decrease in the viability of IOMM-Lee and CH157 cells following treatment with maytansine, as illustrated in **Figure 2A**. The IC30 and IC50 values of maytansine for IOMM-Lee were 0.17 ± 0.04 nM and 0.26 ± 0.06 nM, and for CH157 were 0.20 ± 0.01 nM and 0.31 ± 0.01 nM, respectively, as depicted in **Figure 2B**. Maytansine demonstrated a potent inhibitory effect on the malignant meningioma cell lines IOMM-Lee and CH157, as indicated by IC50 values in the picomolar range.

Subsequently, to assess the impact of maytansine on the radiotherapy of meningioma cells IOMM-Lee and CH157, standard clonogenic survival assays were conducted on 6-well plates using IC30 dosage of maytansine. The results revealed a substantial decrease in the number of colonies when maytansine was administered in conjunction with radiotherapy, as shown in **Figure 2C**. Furthermore, the combination treatment of maytansine and radiotherapy was evaluated for its effect on apoptosis in meningioma cells. Flow cytometry analysis was conducted to evaluate the staining of Annexin V-FITC/PI, which demonstrated a substantial elevation in the apoptosis rate of meningioma cells when treated with the combination approach compared to monotherapy. The apoptosis rates observed in different treatment groups were as follows: For IOMM-Lee cells, the combined therapy group showed an apoptosis rate of 51.7 ± 0.7%, while the radiation alone and maytansine alone groups demonstrated apoptosis rates of 32.7 ± 6.5% and 9.6 ± 2.0%, respectively. In CH157 cells, the apoptosis rates were 56.3 ± 1.7% for combined therapy, 27.6 ± 0.9% for radiation alone, and 18.4 ± 0.2% for maytansine alone. This observation was illustrated in **Figure 2D**. These results indicated that maytansine had a notable sensitizing effect on meningioma cells in an *in vitro* setting.

### Maytansine-treated meningioma cells exhibited altered transcriptional profile

**Figure 3:**
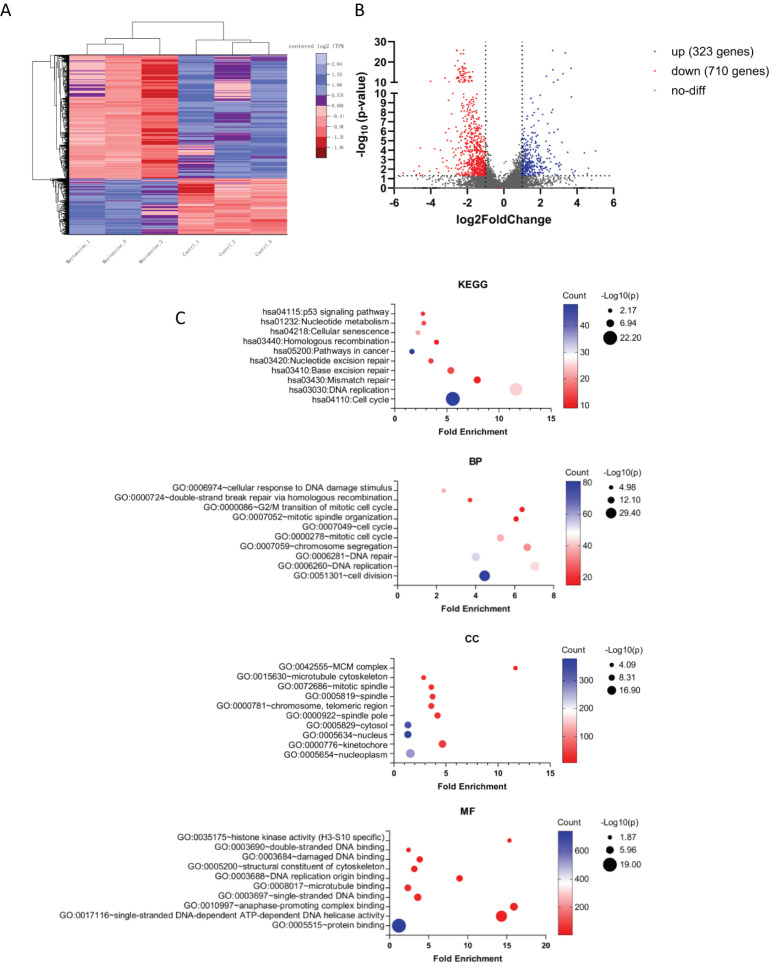
RNA-seq transcriptomic analysis of maytansine on IOMM-Lee cell line *in vitro*. **(A)** The heatmap illustrates genes showing significant differential expression (qValue < 0.05 and |Log2 fold-change| > 1). The control and maytansine treatment samples form clusters, with genes categorized into two main subsets: upregulated genes (blue) and downregulated genes (red). **(B)** The volcano plot displayed significantly differentially expressed genes. Blue dots indicated upregulated genes (up), red dots indicated downregulated genes (down), and gray dots represented genes with no significant change (no-diff). **(C)** Performing Kyoto Encyclopedia of Genes and Genomes (KEGG) analysis and Gene Ontology (GO) analysis on differentially expressed genes, including Biological Process (BP), Cellular Component (CC), and Molecular Function (MF), and reporting the relevant items for each category.

Investigating the molecular mechanisms underlying the synergistic effects of maytansine in radiotherapy enhancement, we performed transcriptomics analysis through RNA sequencing. IOMM-Lee cells were exposed to maytansine for 24 hours, followed by cell harvesting for RNA extraction. Our analysis revealed alterations in the expression levels of 1,033 genes, with 323 genes up-regulated and 710 genes down-regulated (**Figures 3A, B**). Previous studies had validated the efficacy of maytansine as a potent microtubule-targeting agent capable of inducing mitotic arrest and effectively eradicating tumor cells at sub-nanomolar concentrations. In our research, we performed Gene Ontology (GO) and Kyoto Encyclopedia of Genes and Genomes (KEGG) analyses on 1,033 differentially expressed genes (DEGs) using the DAVID database. the KEGG enrichment analysis identified DEGs in pathways associated with the cell cycle and DNA damage repair responses (**Figure 3C**). Likewise, The GO enrichment analysis unveiled significant enrichment in key processes such as the cell cycle, DNA repair, and microtubule binding (**Figure 3C**). Our RNA sequencing analysis of the DEGs demonstrated that maytansine can augment the sensitivity of radiotherapy by modulating cell cycle progression and DNA damage repair mechanisms.

### Maytansine augmented IR-induced DNA damage in meningioma cells

**Figure 4:**
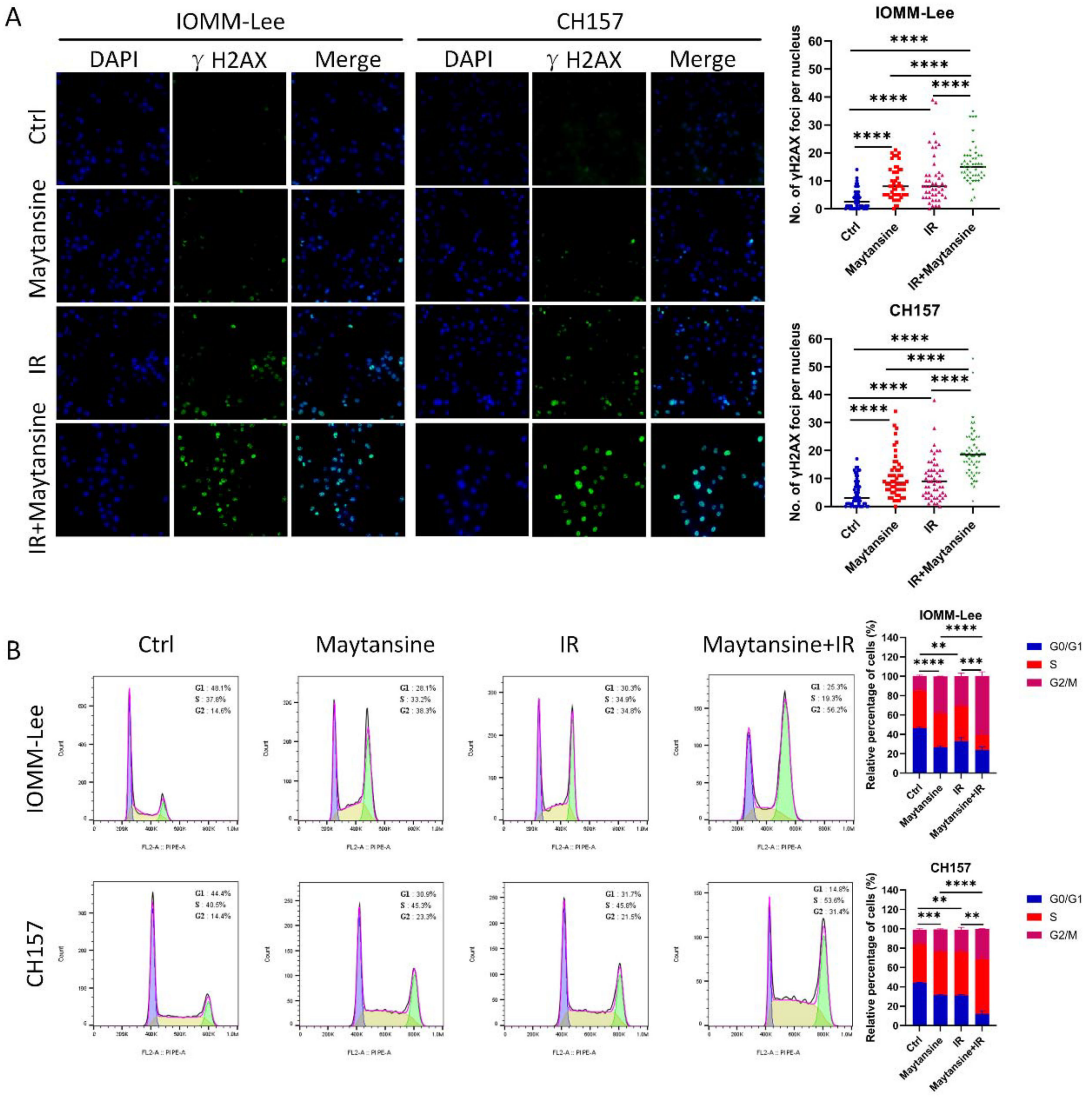
Maytansine augmented IR-induced DNA damage and induced cell accumulation in the G2-M phase of the cell cycle. **(A)** Representative images of IOMM-Lee and CH157 cells in γH2AX foci-based assays, which measure extent of DNA damage. Cultures were exposed to Maytansine followed by 2 Gy radiation therapy 4 hours later. γ-H2AX staining was conducted 24 hours post-treatment, and the average number of γ-H2AX foci per cell nucleus was quantified. **(B)** Cell cycle analysis was conducted using flow cytometry to assess the proportion of IOMM-Lee and CH-157 cells in the G0/G1, S, and G2/M phases of the cell cycle through propidium iodide (PI) staining. Representative histograms illustrating the cell-cycle distribution across four groups (control, maytansine, IR, and Maytansine+IR) were generated. The data represented the mean ± SD from three independent experiments. Statistical significance was assessed using a two-tailed Student’s t-test. Significance levels were denoted as **p < 0.01, *** p< 0.001, and **** p< 0.0001.

To evaluate the impact of maytansine on DNA damage and repair, the expression of γ-H2AX was evaluated via immunofluorescence in CH157 and IOMM-Lee cells as a marker of DNA double-strand breaks (**Figure 4A**). The cells were pretreated with maytansine for 4 hours before exposure to 2 Gy of radiation. γ-H2AX staining was conducted 24 hours post-treatment, and the average number of γ-H2AX foci per cell nucleus was quantified. Treatment with maytansine alone resulted in significant alterations in γ-H2AX levels. Consistently, radiation led to increased γ-H2AX levels 24 hours later. Notably, combination therapy markedly heightened the expression of γ-H2AX in both cell lines, suggesting that maytansine impedes the repair of radiation-induced DNA double-strand breaks.

### The combination of maytansine and IR induced cell accumulation in the G2-M phase of the cell cycle

The G2/M phase had been identified as the most radiation-sensitive stage in the cell cycle, followed by the G1 phase. The late S phase had been found to be the least sensitive. Research had shown that synchronization of the G2/M phase can increase cell sensitivity to IR. To delve deeper into the potential mechanism of maytansine-mediated radiosensitization, the influence of maytansine on cell cycle regulation was investigated. The cells were pretreated with maytansine for 4 hours before exposure to 2 Gy of radiation. Six-hour after radiotherapy, flow cytometry was utilized to assess the cell cycle distribution of meningioma cells. The cell cycle analysis revealed distinct G2/M phase distributions across different treatment groups: In IOMM-Lee cells, the combined therapy resulted in a G2/M phase proportion of 60.7 ± 4.2%, significantly higher than that observed in IR (31.0 ± 3.3%) and maytansine (38.5 ± 0.4%) treatment groups. Similarly, in CH157 cells, the combined therapy demonstrated a G2/M phase proportion of 31.4 ± 0.1%, compared to 22.9 ± 2.3% for IR and 22.8 ± 0.6% for maytansine alone. (**Figure 4B**).

### Maytansine enhanced IR sensitivity *in vivo*

**Figure 5:**
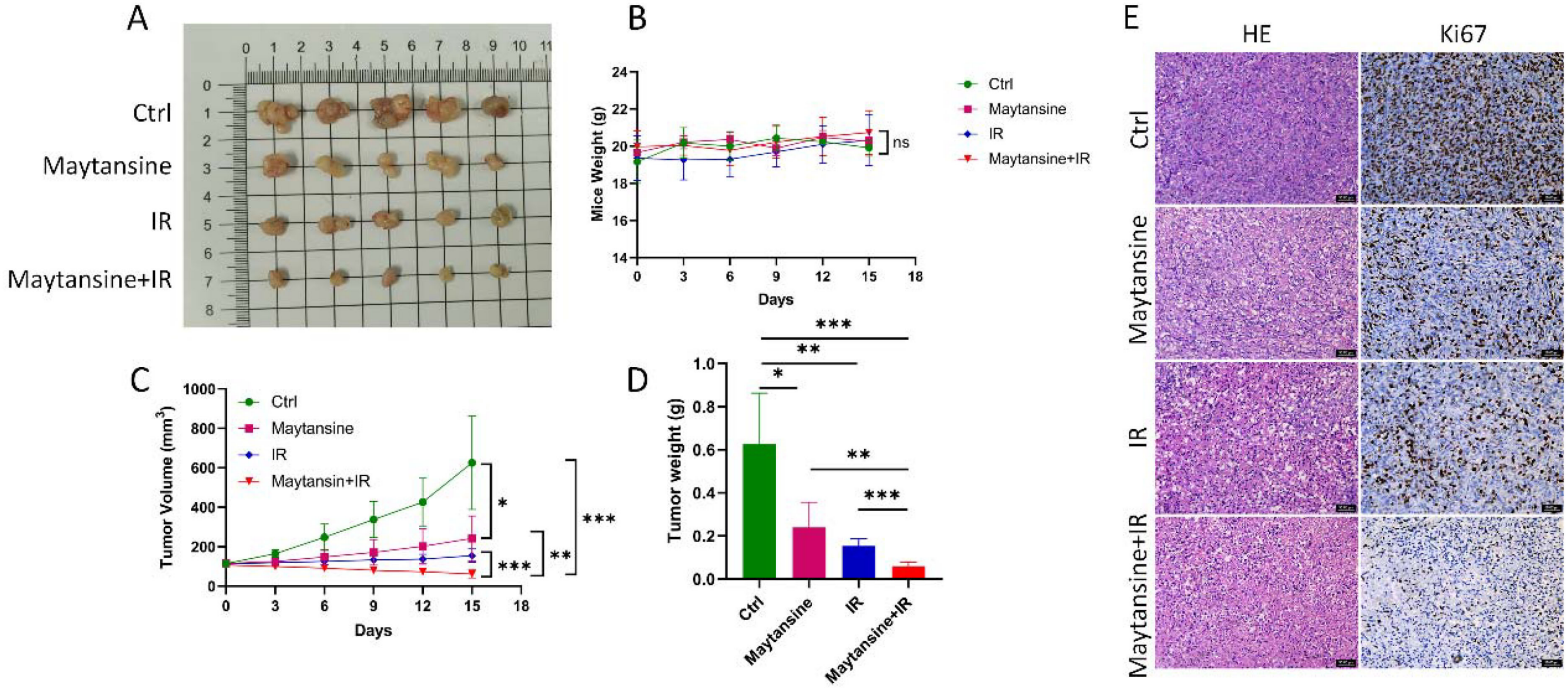
Maytansine enhanced IR sensitivity *in vivo*. A-D. displayed macroscopic images **(A)**, mice weights **(B)**, tumor volumes **(C)**, and tumor weights **(D)** of IOMM-Lee xenograft tumors in each group (n=6). **(E)** Representative hematoxylin and eosin (H&E) staining along with immunohistochemistry (IHC) images of Ki 67 in subcutaneous tumors from all groups. Scale bars: 50 μm. Data are expressed as mean ± SEM. Statistical significance was assessed using a two-tailed Student’s t-test and two-way ANOVA. Significance levels were denoted as *p < 0.05; **p < 0.01; ***p < 0.001.

To investigate the radiosensitivity of maytansine *in vivo*, a subcutaneous transplant tumor model was established in nude mice using the IOMM-Lee cell line. Once the tumor volume reached approximately 100 mm³, the mice were randomly assigned to four treatment groups. Tumor volume and mouse body weight were monitored every 3 days. On the 15th day, the mice were euthanized, and the tumors were removed (**Figure 5A**). No significant differences in mice weight were observed among the four groups (**Figure 5B**). The tumors in the maytansine and IR groups showed decreased tumor weight and volume compared to those in the control group. In contrast, the tumors in the combination treatment group demonstrated even further reductions in tumor weight and volume compared to the other three groups (see **Figures 5C, D**). Hematoxylin and eosin (H&E) stained sections indicated a more conspicuous tumor necrotic region in the combination therapy group, whereas immunohistochemistry revealed decreased Ki-67 expression in the combination therapy group, signifying reduced tumor proliferation capability (**Figure 5E**). These findings confirmed the radiosensitizing effect of maytansine in an *in vivo* setting.

## Discussion

In this study, we identified maytansine as a potent radiosensitizer for meningiomas through a high-content clonogenic survival drug screening of 166 FDA-approved compounds. Our findings demonstrated that maytansine not only exhibited significant cytotoxic effects on meningioma cells but also enhanced their sensitivity to radiotherapy both *in vitro* and *in vivo*. These results highlight the potential of maytansine as a promising therapeutic agent for improving radiotherapy outcomes in meningioma patients.

The identification of maytansine as a potent radiosensitizer for meningiomas holds significant therapeutic implications, particularly for patients with high-grade or recurrent meningiomas who often exhibit resistance to conventional therapies. Our preclinical findings demonstrated that maytansine not only exerted potent cytotoxic effects on meningioma cells but also significantly enhanced their sensitivity to radiotherapy through mechanisms involving cell cycle arrest, DNA damage augmentation, and impaired DNA repair. These results suggest that maytansine could be repurposed as a radiosensitizer to improve radiotherapy outcomes in meningioma patients.

Meningiomas, especially atypical and anaplastic subtypes, frequently develop resistance to radiotherapy, leading to poor clinical outcomes. Maytansine’s ability to enhance IR-induced DNA damage and impair repair mechanisms provides a promising strategy to overcome this resistance. By synchronizing cells in the radiation-sensitive G2/M phase and exacerbating DNA damage, maytansine could significantly improve the efficacy of radiotherapy. The combination of maytansine and IR resulted in a marked reduction in clonogenic survival, increased apoptosis, and enhanced tumor control in both *in vitro* and *in vivo* models. These synergistic effects highlight the potential of maytansine as an adjunct to radiotherapy, potentially allowing for lower radiation doses to achieve therapeutic effects, thereby minimizing radiation-related toxicity.

Insufficient drug treatment options currently existed for meningiomas. Clinical practice lacked drugs specifically designed to enhance the radiosensitivity of meningiomas. In this study, a high content clonogenic survival drug screening was utilized to identify potential radiosensitizing agents for meningiomas. The practicality and sensitivity of this approach had been validated by Lin SH and colleagues, who had also introduced refinements (19, 20). Our research demonstrated that maytansine not only exhibited a significant inhibitory effect on meningioma cells at the pM level but also acted as a robust radiosensitizer. These findings hold substantial clinical relevance in the context of improving the radiosensitization of meningiomas.

Maytansine is a potent microtubule-polymerization inhibitor with a mechanism of action akin to vincristine. By primarily halting cell mitosis, it exerts anti-tumor effects. Notably, it’s *in vitro* anti-tumor efficacy surpassed that of vincristine by 20-100 times and paclitaxel by 24-270 times (21, 22). As a result, maytansine has been proved effective against melanoma, liver cancer, breast cancer, multiple myeloma, lung cancer, and several other malignancies (23–27). Despite its robust anti-tumor properties, clinical utilization of maytansine remains limited due to a narrow therapeutic window, significant side effects, and poor water solubility (28–30).

Efforts in current research regarding maytansine primarily focus on targeted modifications to enhance its tumor specificity, increase tumor tissue distribution, and reduce normal tissue exposure, thereby mitigating toxicity. An example of this approach is the development of antibody-drug conjugates (ADCs) for cancer treatment, demonstrating significant clinical efficacy with minimal adverse effects. FDA-approved ADCs like gemtuzumab ozogamicin for acute myeloid leukemia (31, 32), trastuzumab emtansine for chemotherapy-refractory or advanced HER2-positive breast cancer (33, 34), and brentuximab vedotin for recurrent Hodgkin’s lymphoma (35, 36) had been successfully utilized. Notably, there is currently no established ADC therapy for meningiomas in clinical practice. Transforming maytansine into a targeted therapeutic agent for meningiomas could not only serve as a singular treatment for meningiomas but also be utilized as a radiosensitizer in conjunction with radiotherapy, enhancing the radiotherapeutic outcomes for patients with meningiomas.

Given the high expression of somatostatin receptors (SSTR) in meningiomas, specifically the SSTR2a subtype found in 90% of cases (37–39), SSTR2 emerges as an ideal target for meningioma therapy. The use of Gallium-68 (68Ga) - DOTATATE (38, 40), a contrast agent aimed at SSTR2, had proven valuable in clinical imaging of meningiomas. Furthermore, 177Lu DOTATATE (41, 42), another agent that targets SSTR2, had been employed in radionuclide therapy for meningiomas. Despite these advancements, effective radiosensitizing drugs for meningiomas remain lacking. Recent investigations by Youngblood MW et al. had identified docetaxel as a promising candidate capable of targeting the aggressive methylation profile in high-risk meningiomas and functioning as a radiosensitizer (43). Additionally, Chen K et al. (44)had developed an anti-SSTR2 ADC for targeted therapeutic treatment of meningiomas. Flow cytometry analysis indicated a binding rate exceeding 98% for the anti-SSTR2 monoclonal antibody with meningioma CH157-MN cells, while remaining below 5% in normal arachnoid AC07 cells. *In vivo* imaging via the *In Vivo* Imaging System (IVIS) displayed the specific targeting and accumulation of cy5.5-labeled ADC in xenograft meningiomas with minimal presence in healthy organs. Pharmacokinetic assessments and histological analysis affirmed the stability and low toxicity of the ADC. Both *in vitro* and *in vivo* experiments corroborated the efficacy of the anti-SSTR2 ADC in tumor growth inhibition. However, challenges remained as the antibody requires humanization, and the ADC formulation awaits clinical trial validation, mandating further exploration to ascertain its full therapeutic potential.

## Conclusions

Overall, our study primarily focused on the screening of radiosensitizers for meningiomas through high-throughput methods. Among the pool of 166 potential drugs for meningiomas, maytansine exhibited significant radiosensitizing effects at pM concentrations, indicating promising potential for the development of radiosensitizers to meningiomas. However, this study had certain limitations, particularly the lack of involvement in ADC modification of maytansine. Therefore, our future research direction aims to develop ADC drugs that can be clinically implemented for radiosensitization of meningiomas.

## Data Availability

The sequencing data generated in this study have been deposited in the NCBI Sequence Read Archive under accession number PRJNA1234314.
